# A Layered Middleware for OT/IT Convergence to Empower Industry 5.0 Applications

**DOI:** 10.3390/s22010190

**Published:** 2021-12-28

**Authors:** Lorenzo Patera, Andrea Garbugli, Armir Bujari, Domenico Scotece, Antonio Corradi

**Affiliations:** Department of Computer Science and Engineering (DISI), University of Bologna, Viale Risorgimento 2, 40136 Bologna, Italy; lorenzo.patera@unibo.it (L.P.); andrea.garbugli@unibo.it (A.G.); armir.bujari@unibo.it (A.B.); domenico.scotece@unibo.it (D.S.)

**Keywords:** Industry 5.0, smart manufacturing, OT/IT integration, cloud continuum, machine-to-machine, cyber–physical systems

## Abstract

We are still in the midst of Industry 4.0 (I4.0), with more manufacturing lines being labeled as smart thanks to the integration of advanced ICT in Cyber–Physical Systems (CPS). While I4.0 aims to provision cognitive CPS systems, the nascent Industry 5.0 (I5.0) era goes a step beyond, aiming to build cross-border, sustainable, and circular value chains benefiting society as a whole. An enabler of this vision is the integration of data and AI in the industrial decision-making process, which does not exhibit yet a coordination between the Operation and Information Technology domains (OT/IT). This work proposes an architectural approach and an accompanying software prototype addressing the OT/IT convergence problem. The approach is based on a two-layered middleware solution, where each layer aims to better serve the specific differentiated requirements of the OT and IT layers. The proposal is validated in a real testbed, employing actual machine data, showing the capacity of the components to gracefully scale and serve increasing data volumes.

## 1. Introduction

The emergent fifth industry revolution, also known as Industry 5.0 (I5.0), aims to establish value chains spanning heterogeneous industrial domains, enhancing re-use, increasing production flexibility, and exhibiting resiliency in times of disruption [1]. I5.0 is a long-term vision taking us beyond the I4.0 era, by fostering seamless cooperation and coordination of processes, building circular and sustainable value chains.

Sensing technology, big data, and artificial intelligence (AI) have proven viable at automating, managing, and optimizing a wide range of non-industrial processes, and, recently, this practice is expanding in the industrial domain [2]. The current manufacturing landscape comprises heterogeneous machines and production facilities capable of autonomous message exchange, generating data at an ever-increasing speed, and all data could provide useful information and could be used proactively for optimized control and business-related purposes [3]. This capability could bring fundamental improvements to the industrial processes in manufacturing, engineering, supply chain, and life cycle management [4].

However, a big obstacle in achieving this goal, especially for SMEs, is the obsolete and rigid separation between technologies that characterize departments involved in product manufacturing (working machines and production lines) and departments committed to managerial tasks [5]. Indeed, industrial automation has taken a conservative approach, opting for separation between the Operation and Information Technology domains (OT&IT). OT consists of systems that monitor and control physical processes that manage automated manufacturing, and the associated applications are typically safety-critical and real-time, embodying extra non-functional properties, such as bounded latency, reliability, and compliance with industry-specific safety and security standards [6,7]. Until now, IT technologies, such as cloud/edge computing, Service-Oriented Architectures (SOA), and virtualization, have been exploited in industrial applications only in a limited way, i.e., only in contexts where very stringent requirements were not needed. However, it is becoming obvious that I5.0 will have a very significant impact only with a full convergence of OT/IT that will push for the deep joint exploitation of most recent computing and communication technologies.

Zooming in on the OT layer, a wide range of protocols co-exist which are incompatible with one another, leading to fragmentation that makes it difficult to provide a coherent and consolidated view of the assets and processes. To this end, different proposals have emerged, with OPC Unified Architecture (OPC UA) factually becoming the de facto interoperability standard [8].

This work presents the design of an OT/IT convergence solution, and a ready to use software solution, exploiting the edge computing paradigm to enable fast and reliable data sharing on the OT/IT boundary. Our proposal relies on a two-layered middleware approach, where each layer aims to best face and comply with the different requirements of the OT and IT layer, respectively, and is also capable to provide a better synergy of the two layers. In particular, we adopt OPC UA as the interoperability layer of systems and devices, best suited to fulfill stringent Quality of Service (QoS) requirements of OT layer traffic. A Gateway component, deployed on the edge computing fabric, defined to handle the coordination of layers at the OT/IT boundary, is capable of conveying OT data towards the IT layer with reliability and security. To this end, we rely on Apache Kafka, a Message-oriented Middleware (MoM) equipped with a rich ecosystem of plugins, capable of providing differentiated QoS to OT flows.

The contribution of this work is three-fold: (i) we present a practical solution for the OT/IT convergence problem (ii), we present the implementation details of a two-layered middleware approach best fitting the needs of the OT/IT layers, and (iii) we validate the proposal in a real testbed. The remainder of this article is structured as follows: Section 2 provides a concise survey on the OT/IT problem along with some background information on the technological ecosystem adopted in this work. Section 3 presents some prior research effort in the context, identifying the gaps. Next, Section 4 provides an overview of the main functional building blocks and interaction points, while Section 5 presents the experimental assessment. Finally, Section 6 draws the conclusions.

## 2. Background

This section provides some preliminary background on the OT/IT convergence, so discussing the benefits and technology strategies needed to achieve this goal. Next, we provide an overview of two main building blocks of our proposal, reasoning about their pros/cons, identifying potential synergies. We detail the OPC UA standard architecture as a necessity of the OT reality, and also an IT view of the Kafka middleware since those technologies are the base of our proposal.

### 2.1. Cloud-to-Thing Continuum for OT/IT Convergence

In traditional industrial networks, the Operational and Information Technology layers co-exist as separate entities to serve different purposes. On the one hand, the OT layer involves components interacting directly with the physical machines and extracting data from them. On the other hand, the IT layer typically has more relaxed requirements than the OT layer, since it is composed of computer systems that create, transmit and safely store data with almost no time constraints.

Recently, the market penetration of Industrial IoT (IIoT) networked devices, equipped with sensing and communication capabilities has enabled companies to connect devices on the plant floor, developing cyber–physical systems capable of generating and collecting data throughout the entire industrial space [4]. That has also contributed to a renewed interest in the OT/IT convergence topic, identified by Gartner [9] among the top areas of investment in the near term.

The benefits of an OT/IT convergence solution have long been acknowledged by academia, and, very recently, taken inside the umbrella of several industry standards and bodies. As an example, the 5G-AICA industrial consortium recognizes edge computing as a pillar technology which could, among other benefits, help blur the strict OT/IT separation in order to gain flexibility and intelligence [10].

**Figure 1:**
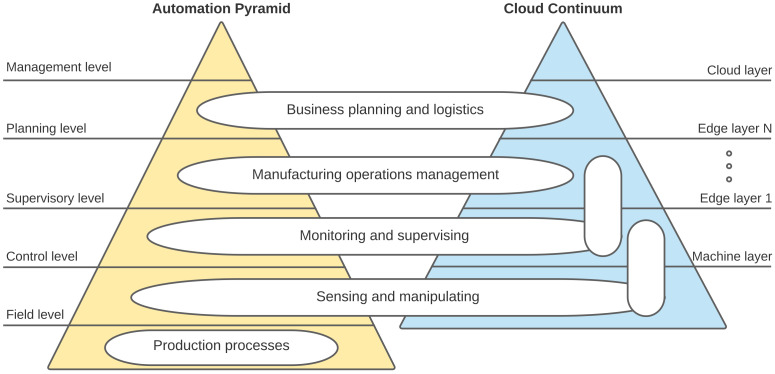
Automation pyramid remapped on Cloud continuum representation.

The blurring of the OT/IT strict boundary would open the door to the next-generation Industry 5.0 industrial applications, allowing for fine-grained monitoring and control of individual assets and processes via Digital Twin technology [11,12]. In the IIoT context, in particular in production manufacturing plants, cloud/edge computing is considered a relevant opportunity that can significantly contribute to blurring the current separation of OT&IT domains through the design of edge nodes where compute/storage/networking functionalities could converge. As shown in Figure 1, several hierarchical layers of edge nodes with different capabilities can be deployed, distributing the resources along to support the execution of industrial applications and their data storage, thus giving rise to a more fluid model identified as Cloud-to-Thing Continuum (C2TC) [6]. It is clear, however, that just introducing support for the execution of industrial applications at the edge nodes is not sufficient. Seamless integration at all the levels of the infrastructure (cloud and edge) is needed to ensure the application QoS specifications.

In this context, edge computing plays a crucial role in enabling the design and implementation of novel distributed control functions with parts that are hosted on the edge nodes located in the production plant premises and close to the controlled sensors/actuators, primarily to increase reliability and decrease latency [13]. This edge-enhanced cloud architecture provides several benefits compared to a pure data center-based approach: application latency is reduced because of vicinity to end-points; inter-domain traffic is diminished because, for example, Multi-access Edge Computing (MEC) nodes stay in the telco operator network; sensitive information/processing (e.g., of monitoring data related to the manufacturing process that can reveal competitive advantages) can be maintained at industrial edge gateways in the premises of end-points, while global status visibility can be employed, e.g., when needed for global machine learning optimization, by interacting with pure data center-based cloud resources [14].

### 2.2. OPC Unified Architecture

Ethernet technology has seen steady growth in adoption in the industrial automation sector, leading to overcome the well-known Fieldbus family of the technology, becoming a de facto standard in the OT domain. A multitude of Ethernet variants has been developed and deployed over the years: PROFINET, EtherCAT, and Modbus-TCP [4]. However, these technologies are incompatible with one another, leading to fragmentation at the OT layer, making it difficult to provide a coherent and consolidated view of assets and processes. For this reason, one of the most promising protocols in the context of Industry 5.0 is OPC Unified Architecture [15], proposed as a platform-independent standard facilitating interoperability among vendors.

At first, the standard adhered to a Client/Server paradigm: an OPC UA server provides access to data and functionality structured in an object-oriented information model, while clients interact with the information model via a set of standardized services. Communication takes place in this setting, by following the classical request-response model. This interaction does not suit our application needs as: (i) it introduces strong coupling between different system parts, and (ii) this communication model impedes is not suited to meet the performance required by a hard real-time system.

For this reason, Part 14 of the OPC UA specification defines an extension of OPC UA based on the Publish/Subscribe (Pub/Sub) communication paradigm [16]. In this communication model, an application can play the role of either publisher or subscriber (even both sometimes), where the former is the source of data, while the latter consumes that data.

The communication between publishers and subscribers is message-based: the publisher sends the messages to a message-oriented middleware, without taking into account the possible number of subscribers. Likewise, subscribers show interest in one or more types of data without having any specific information about the publishers. The Pub/Sub model is best suited for applications where location independence and scalability are required.

The MoM is a well-known infrastructure used to send and receive messages in distributed systems, pursued by OPC UA, suited to many use cases in the industrial domain. More specifically, OPC UA Pub/Sub supports two different MoM architectures:

1. Broker-based: the core component of the MoM infrastructure is a message broker. Subscribers and publishers use standard messaging protocols like AMQP or MQTT to communicate with the broker [17], with messages being published to specific queues (e.g., topics, nodes) exposed by the broker. The broker is tasked with translating messages from the messaging protocol of the publishers to the messaging protocol of the subscribers.
2. A brokerless form, where the MoM is the network infrastructure, capable of routing datagram-based messages, and subscribers and publishers use a datagram-oriented protocol like UDP. The broker-less model is intuitively the one embodying the best performance, and therefore best suited to fulfill our system requirements. Addressing the needs of this deployment model, the specification defines a custom UDP-based protocol, called UADP [16] which relies on a multicast scheme for communication among parties.

Focusing on the implementation of the brokerless form, a subscriber entity registers to a multicast group represented by an IP address in a special range. Data sent to this address are forwarded to all members of the group. This delegates a large part of the publisher’s complexity to the existing IP network infrastructure (router, switches, and so on).

While OPC UA is the most significant IoT protocol proposed to address the fragmentation and communication needs at the OT layer, it does not fully address the needs of the IT layer. In this context, we are in need of solutions and frameworks capable of handling high-throughput data transfer in a reliable and secure manner, while these features are not the primary concerns of OPC UA. In the following, we present our proposed framework.

### 2.3. Apache Kafka

Apache Kafka is an open-source MOM that enables many-to-many communication via a Pub/Sub paradigm, well suited in scenarios where scalable and loosely coupled systems need to interoperate. The main components are producers and consumers, where producers publish messages or batches of messages on an abstraction of a communication channel called topic, and consumers can read the messages by subscribing to a topic, thereafter receiving the published information.

Topics use extensively the partition abstraction that can be seen as an infinite log file with no immediate flush on disk, allowing very efficient I/O messaging. Kafka has stronger ordering constraints compared to a “traditional” messaging system, guaranteeing a total ordering on all messages belonging to the same partition. Moreover, it allows overcoming the traditional publish–subscribe mechanisms, by providing abstractions for consuming messages with different semantic and replication degrees, using consumer group id identifiers. By taking advantage of this functionality, a developer can use the same consumer group id to do implicit load balancing between servers or can use a different one for broadcast messages [18].

Apache Kafka is supported by Confluent Inc. and by a growing community of developers that have created an ecosystem of interoperable tools or plugins. The Kafka Connect add-on permits to extend the platform with the plugins and to import/export data from/to almost any popular database, processing platform, and real-time application [19], making Kafka an interoperable and extensible middleware solution.

In the following, we discuss some prior efforts, addressing the OT/IT convergence problem, while at the same time we discuss related work within that guideline, motivating the rationale behind our approach.

## 3. Related Work

This session compares different relevant proposals so to show the state of the art of support for the convergence of OT/IT layers.

Indeed, this topic has been covered by authoritative standardization bodies such as the International Society of Automation (ISA) [20] and the International Electrotechnical Commission (IEC) [21]. As an example, the multi-layer IEC 62264 standard based on the ISA-95 specifications [22] defines an information model exchange framework enabling the integration of applications running in business and operational departments. Enterprises complying with the standard can define interfaces between control and business functions, allowing them to make informed decisions on data to exchange so that costs and risks can be kept low in case of implementation errors.

Among other authoritative standards is the European Reference Architectural Model Industry 4.0 (RAMI 4.0) [23], advocating for tight coordination of IT and OT. To this aim, RAMI 4.0 proposes a high-level reference architecture addressing the broad spectrum of Industry 4.0 scenarios. In particular, the reference communication layer of the RAMI 4.0 connects the concept of I4.0 to the OPC UA standard, electing the former as the one and only choice guaranteeing interoperability at the OT [24].

In [25] the authors present a cloud-based framework designed for smart factories. The system proposes an integration method between a monitoring system and an optimization system, both distributed as cloud services. In particular, monitoring data can be collected from different sources (e.g., sensors and operators) and then sent to the services via the REST API. The work presents an appealing case study, relying on the cloud to collect and optimize manufacturing processes. Our work focuses on OT/IT integration, enabling granular and secure data collection, enabling a wide range of potential use cases.

In [26] the authors show the need for the adoption of the edge computing paradigm in the industrial context, outlining the lack of a reference model which would support the classification of current and future research. To this end, the authors attempt to fill in this gap, proposing the Reference Architecture Model Edge Computing (RAMEC) for use in the context of industrial automation. As a result, the authors identify 210 distinct views for the Edge Computing paradigm in the manufacturing domain. Our novel infrastructure perfectly fits the hierarchy levels outlined in the article, varying from the “Deep Edge” (data uniform representation) to the “Private Edge” (data gathering in a private middleware deployed in the firm). In this regard, we present a practical implementation, assessed in a real testbed scenario.

In [27] the authors propose an experimental methodology to investigate the impact of QoS parameters on the communication delay from the production line to the Cloud and vice versa. In this study, the OPC UA standard is adopted at the OT layer, showing that industrial IoT gateways solutions based on OPC UA have a great impact on the delivery metrics. Similar to our approach, the authors in their work adopt OPC UA as a platform-independent protocol, while they rely on the Cloud for data management and analytics. We move a step further in this direction, demonstrating a practical integration of edge computing to address the OT/IT convergence problem without strict Cloud-reliance.

In [28] the authors argue that, despite OPC UA Client/Server architecture being the de facto standard for several use-cases in the upper levels of the automation pyramid, is not suitable for the communication in lower levels, e.g., control-to-control and field level traffic. In addition, the authors introduce the OPC UA Pub/Sub architecture, identifying four different combinations of OPC UA communications, evaluating and comparing their applicability on a range of factory automation use-cases.

In the same direction as the prior work, the authors in [8] present OPC UA over TSN as a vendor-independent technology, identifying this technology as the first and only candidate for establishing a holistic communication infrastructure from the sensor to the cloud. To do so, the authors compare OPC UA over TSN with the most significant Ethernet-based M2M Fieldbus systems—known as Ethernet real-time protocols, demonstrating the benefits of the former.

In our previous work [29] we proposed a multi-layer architecture to monitor legacy industrial equipment during their operations inside customer plants. The proposed architecture provides near-real-time data gathering powered by two Apache Kafka installations, in OT and IT, respectively. In the proposal, several hmi-forwarders—dedicated software components acting as both adapter and gateway—interface directly with Modbus-TCP machinery and export data in the OT Kafka instance. From here on, the data is then forwarded to the specific Kafka topic at the IT layer.

Preliminary collected results were encouraging: the components of the platform maintain a constant delay at the increase of the number of messages in transit in the system, thus confirming the scalability of the architecture. However, the latency assessed in the upper layer is about 70 ms, completely inadequate for M2M communication and incompatible with OT needs.

This paper improves our prior proposal in several different directions. First, it splits the functions of the hmi-forwarders component into two distinct ones: adapters and gateways. This decoupling allows for a pluggable machine layer that can be enriched and support additional languages on the shop floor. That also allows the Gateway to be deployed on an edge computing layer, so decoupling OT- and IT-related functionalities. At the same time, we intend to define a support capable of enabling and expressing the necessary different convergence requirements of IT and OT layers. In addition, we state that the proposed solution is going to be assessed in a real testbed scenario, by using real machine data.

## 4. Our Proposal

Herein, we describe the approach taken to effectively blur the OT/IT boundary, so enabling fast, reliable, and secure operational OT data exchange towards the IT layer. As anticipated, the approach relies on a Gateway component that resides on the OT/IT boundary and a two-layered middleware solution aimed at fulfilling both the functional and the non-functional requirements of each layer.

### 4.1. System Components and Integration

Our proposal relies on the OPC UA Pub/Sub for machine-to-machine (M2M) communication at the OT layer and uses Apache Kafka, a high-throughput, low-latency Message-oriented Middleware (MoM), for data gathering from multiple OT sites towards the IT domain. Though in principle, the OPC UA standard allows to reach and convey data above the OT layer to the upper layers—SCADA, IT, Cloud, it is mainly a low-level interoperability protocol allowing fast transmission of data. At the IT layer, it is the Kafka MoM that permits the handling of large volumes of data in a secure and reliable manner, while at the same time, presents an extensible framework with a rich ecosystem of tools for IT.

**Figure 2:**
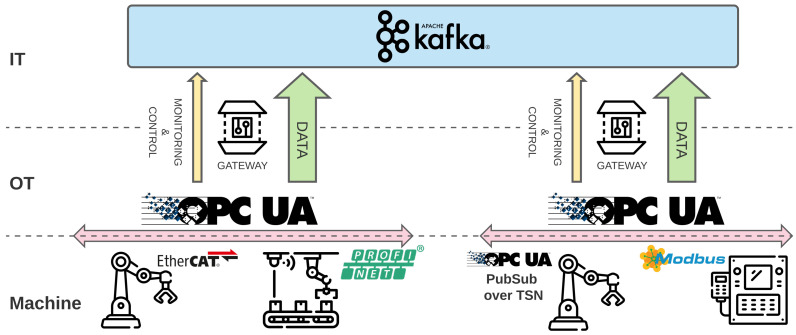
Architecture overview diagram.

Figure 2 shows a schematic representation of the main components of the architecture. The dotted line denotes the separation of the OT, IT, and Machine areas. In the Machine layer, the assets use several low-level and heterogeneous protocols some of which adhere to standard specifications with open-source implementation (e.g., OPC UA over TSN), meanwhile the majority do not generally interoperate, are closed source, and, most times, proprietary (e.g., EtherCAT, PROFINET, Modbus-TCP) [30]. In our proposal, the area on top of the assets, namely the OT, acts as a homogenization layer, by abstracting away from the upper layers the technical details of the specific protocols. The OT layer has a pluggable architecture, so to allow specific adapter components to be added dynamically into the infrastructure.

After configuration, the adapter initiates the data collection according to the machine-specific language, exposing the machine information model via the common OPC UA standard. As anticipated, the rationale of that choice is to have a representation of the information common among the machine and the upper layers. We point out that our architecture can support different adapter deployment strategies, depending also on the computational resources available on the specific industrial asset: if the machine has enough resources, the adapter can be directly deployed on it; otherwise, the adapter can be deployed elsewhere and is connected to the machine via the network fabric.

On top of the OPC UA protocol, we use the OPC UA Pub/Sub specification for message exchange inside the single shop floor. In the figure, the shop floor is depicted as an arrow above the machinery. Herein, heterogeneous traffic needs to co-exist and can vary from safety-critical control traffic to best-effort ones. In practice, data are gathered via a Gateway component which listens on OPC UA Pub/Sub endpoints and sends data to the Kafka MOM. Gateways are customized via configuration files that specify machine addresses and registers that must be manipulated and re-exposed on Kafka topics. In Section 4.2 we report an example of the configuration file.

One goal of the Gateway is to differentiate between heterogeneous flows, namely raw sensor data and data deriving from monitoring processes on the shop floor. The former represents the information exposed by the industrial machine, containing data regarding its internal state. Additionally, the monitoring flow comprises the data and metrics related to networks, industrial processes, etc., supported by Kafka, by providing mechanisms and engineering options.

More specifically, on the producer side, we need to prioritize monitoring and control data traffic. To achieve that, we use different topics and different partitioning levels per data type, where the monitoring and control topics are configured to have a single partition and a higher degree of replication of that partition. This engineering option guarantees a total ordering of sent messages and an enhanced fault tolerance. Concerning the raw sensor data, the topics are configured to have multiple partitions and a lower degree of replication, guaranteeing higher input/throughput rates and lower memory usage.

At the consumer side, we use Apache Kafka differentiated semantics: At-Most-Once, At-Least-Once, and Exactly-Once for the commit management setting [31]; for monitoring and control data we exploit an Exactly-Once semantic; while for the data traffic, we use an At-Least-Once semantic, for faster reading.

Concluding, Apache Kafka supports Access Control Lists [32] via the so-called ACL Authorizers. This feature can be used in industrial settings since data confidentiality is of paramount importance.

Access control allows us to apply fine-grained access policies on the topics, by defining groups of authorized readers and writers and improving the security of the entire infrastructure. On the other side, considering the stringent latency requirements at the OT layer, we assume it is not directly exposed to the external world, hence no particular security mechanisms are in place and the software running in this domain is certified and guaranteed not to pose any threat. These aspects deserve further investigation, and we are currently looking into the adoption of lightweight security mechanisms into our solution.

### 4.2. Bootstrapping the System

To bootstrap the system, one needs to provide some essential configuration parameters, binding the components together and initiating a structured information exchange towards the IT layer. The steps involved are as follows:

- **Configuration**: the Gateway component is issued a structured configuration file, containing the addresses of OPC UA enabled assets, such as IP addresses and multicast network groups on which to register the industrial asset internal state. Other configuration parameters contain information regarding the Kafka endpoints and topics on which to publish messages, QoS level mappings, and their publication frequency. For the sake of clarity, an example configuration file is reported in Listing 1.
- **Discovery**: the Gateway queries the OPC UA server(s) to verify the representation of the data. In this phase, the Gateway also checks if the OPC UA reported registers are coherent to what has been reported in the configuration file.
- **Operations**: once the discovery phase completes with success, the Gateway subscribes to the multicast network groups, starting the flow of messages, which upon reception in a specific protocol dialect, are un/marshaled to a (configurable) JSON representation. Depending on the data type, the messages can be sent on different channels. For instance, the level can be set on high-quality and ordered for “control” flows, guaranteeing fast and reliable delivery, while sensor messages, can be sent with a non-ordered semantic, depending on customer-specific policies.

From this point onwards, we consider that the data are available and can be fetched from the Kafka topics, and that data can be read by multiple consumers, depending on specific access policies.

It is noteworthy to point out that the decoupling of the OT and IT layer, through the use of a lightweight configurable Gateway, enables us to implement advanced control features addressing reliability and scalability in scenarios of high ingress traffic. To this aim, we are currently investigating the design and implementation of a lightweight control and management plane, allowing for the run time deployment of customized coordination and synchronization schemes among the Gateway components.

Listing 1 **Example of JSON configuration file used by the Gateway.**

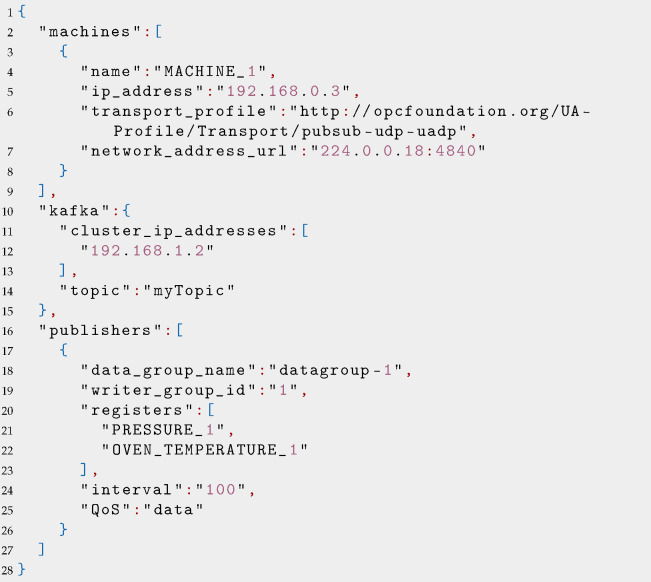

## 5. Experimental Analysis

The goal of the experiment is to validate our architecture so as to show its capacity to work while suiting different constraints in an effective way. We intend to show the capacity to support QoS specifications of low-latency flows at the OT layer, while at the same time assessing the capability of providing high-throughput and quality data to the IT layer. To this end, we have developed a testbed depicted in Figure 2.

### 5.1. Experimental Settings

To fully assess the functional capabilities of our proposal, we have deployed a real testbed of five nodes, hosting different functionalities related to the OT and IT layer and where nodes are connected via a dedicated network consisting of a 1 GB switch. While this network setting may not be as rich as a real deployment scenario, it suffices the purpose of this work, aimed at testing and assessing the functional components of our architecture in an operational environment. For completeness, Table 1 reports the characteristics of the deployed nodes.

Two nodes of the infrastructure are dedicated to traffic simulation. For this part, we rely on some software packages emulating realistic industrial machine traffic, build and developed from scratch starting from actual industrial machinery specifications. More details on this part can be found in a previous paper [29].

More specifically, the first simulator (Node 1) simulates an industrial asset, by exposing its internal operational state via the Modbus/TCP protocol. A Modbus adapter at the machine layer can read and extract the information in a protocol-agnostic format, successively exposing and structuring the machine information by using the OPC UA data model. Finally, the data is transmitted by using the OPC UA Pub/Sub protocol. Let us note that the adapter acts both as a subscriber to and publisher of data depending on the configuration and purpose. Then, the information is available to be received by all other entities present in the network (machines and gateways). A consumer, receiving the data emitted by the first simulator, is deployed at Node 2, where the software implemented represents an OPC UA Subscriber. That subscribes to the first simulator and begins receiving the messages published by the first machine. This behavior simulates a typical sensors-to-controller scenario.

Next, Node 3 hosts the Gateway component where it subscribes to the messages sent by the simulator present in Node 1. These are the same messages received by the simulator in Node 2. Node 4 is a Docker-based Kafka deployment that receives messages produced by the Gateway that acts as a producer. Finally, Node 5 hosts a Kafka consumer, consisting of a custom program that receives messages from specific Kafka topics. The consumer allows us to estimate the transit time that takes a message from Node 1 to a consumer in the IT department of the factory or directly to the Cloud.

In order to accurately measure time, nodes are synchronized by using the Precision Time Protocol (PTP) to extract fine-grained metrics in the time domain. Toward that goal, the node hosting the Gateway is configured as the controller, providing a reference clock for all other entities that participate in the PTP domain, whereas the others act as responders. For additional details on the implementation front, we refer the reader to the public repository containing the source code of the project [33].

### 5.2. Results

To assess the proposal, we measure the message latency from the OT-to-IT layer, under varying traffic regimes.

**Figure 3:**
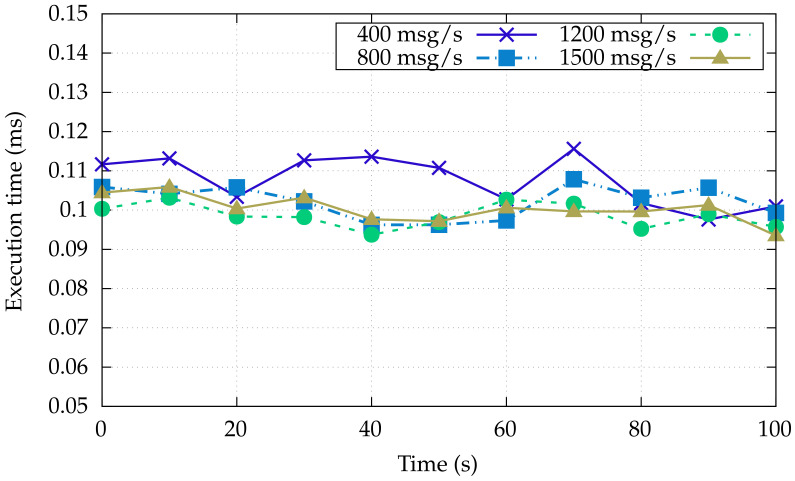
Machine-to-machine communication latency under varying message load of the OT layer.

**Figure 4:**
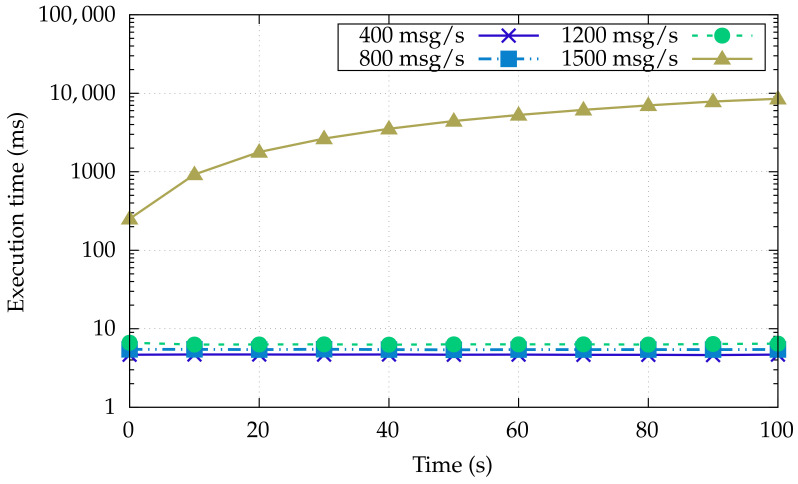
Machine-to-consumer communication latency under varying message load of the IT layer.

Figure 3 shows the latency in the OT level (Node 1 → Node 2), whereas Figure 4 measures the end-to-end latency, that is from the OT layer to the Kafka consumer in the IT layer (Node 5). In both cases, the latency is computed as the time period between the receiving and sending time at the application layer, by sending messages with a different rate, from 400 to 1500 messages per second.

Figure 3 shows that the latency between the two simulated machines remains stable while increasing in the number of messages/second up to 1500 per second. Most importantly, we always observe a sub-millisecond latency, which is the required latency expected at the OT layer, in particular for the communication between different machines or PLCs.

Figure 4 shows the end-to-end latency measured at the IT level for the same message rate above, by exhibiting a latency that is an order of magnitude higher than the one sensed in the OT level. That increase is expected when considering both the number of software components the message must traverse, and the latency introduced by the Kafka MOM features. The latter has been configured to manage the forwarding of the messages to the consumer by imposing a total ordering and ensuring exactly one semantics (single topic/single partition) which is particularly important when conveying safety-critical information from the OT. The effects of the above-mentioned semantics are clearly visible in the 1500 message/second configuration, causing up to an exponential growth in latency. In fact, in this setting, the rate mismatch of servicing input data, marshaling of messages to IT-layer compliant format, and their emission to the respective output queue, creates an increasing backlog of messages over time. This trend gives evidence that not all data and information exchanged in the OT could be sent to IT whenever low latency and no data loss are requirements. To solve that mismatch, the OT layer can be equipped with selective pre-processing capability, specifically using filtering and aggregation, to better coordinate the different layers and to alleviate the burden at the OT/IT bridging point.

**Figure 5:**
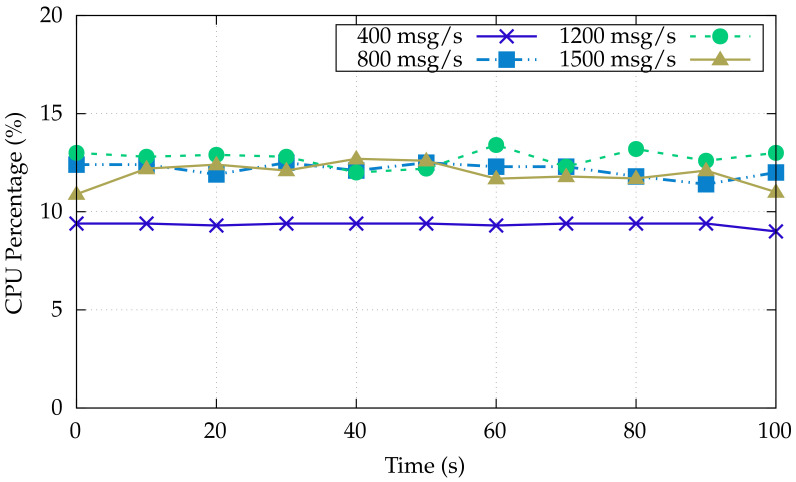
Gateway CPU usage under varying message loads.

That line is also confirmed by data shown in Figure 5, showing the Gateway CPU usage, evidencing an increase in CPU usage trend, augmenting together with the increase of message arrival rate, while still having plenty of resources that could be devoted to other computational tasks.

The experimental results confirm our initial intuition of adopting tailored solutions for the OT and IT layer while relying on an edge computing fabric hosting dedicated functionalities bridging the two worlds. Results at the OT layer show sub-millisecond latencies, satisfying latency requirements for latency-critical traffic at the OT. Similarly, the adoption of Kafka provides greater flexibility at the IT layer, allowing traffic differentiation through a rich set of customizable features.

## 6. Conclusions

Industry 5.0 presents a model for the next level of industrialization, advocating for intelligent supply chains and hyper customization. The integration of data and AI in industrial decision-making is at the core of this new vision, providing the basis for a cognitive shop floor. This vision however is hampesred by missing coordination between the operation and technology domains, demanding an immediate solution.

To this end, we proposed a practical solution addressing the OT/IT convergence problem, by exploiting the edge computing paradigm to blur the strict OT/IT separation. To fulfill the specific needs of OT and IT, we rely on a two-layered MoM approach acting as an interoperability layer at the OT, while, at the same time, enabling fast, homogeneous transfer of large volumes of data towards the IT. The proposal was validated in a real testbed employing actual machine data, evidencing the capability of the components to gracefully scale and serve increasing data volumes.

As future work, we are currently exploring the possibility to introduce a pluggable, lightweight processing mechanism at the Gateway for OT data filtering and aggregation. This feature could be provisioned dynamically and selectively at the edge, alleviating the pressure on the upward path. In this direction, we are exploring the adoption of an event-centric serverless processing model able to dynamically and autonomously scale-out/-in resource usage at the edge.

Complementary to this objective is the study and introduction of a load balancing mechanism at the gateway(s), increasing the reliability and the scalability of the proposal when faced with an increasing traffic pressure originating at the OT layer. Another important direction is that of enabling an IT-to-OT data flow via tailored security mechanisms, opening the door to future cognitive agents residing at the IT layer. 

## Figures and Tables

**Table 1:** Testbed deployment: components, OS, and hardware characteristics.

Name	Component	Operating System	CPU	RAM	Network
Node 1	Machine Simulator 1	Ubuntu 20.04.3 LTS	Intel Core i5-2400 CPU @ 3.10GHz	8 GB	1 Gpbs
Node 2	Machine Simulator 2				
Node 3	Gateway				
Node 4	Kafka Consumer				
Node 5	Apache Kafka	Ubuntu 20.04.3 LTS	Intel Core i5-3470 CPU @ 3.20GHz	16 GB	1 Gpbs

## Data Availability

Not applicable.
